# Gait and Neuromuscular Changes Are Evident in Some Masters Club Level Runners 24-h After Interval Training Run

**DOI:** 10.3389/fspor.2022.830278

**Published:** 2022-06-02

**Authors:** Sherveen Riazati, Nick Caplan, Marcos Matabuena, Philip R. Hayes

**Affiliations:** ^1^Department of Sport Exercise and Rehabilitation, Faculty of Health and Life Sciences, Northumbria University, Newcastle upon Tyne, United Kingdom; ^2^Biomechanics, Rehabilitation, and Integrative Neuroscience Lab, Department of Physical Medicine and Rehabilitation, School of Medicine, University of California, Davis, Davis, CA, United States; ^3^Unique Research Center on Intelligent Technologies (CiTIUS), University of Santiago of Compostela, Santiago de Compostela, Spain

**Keywords:** gait, biomechanics, neuromuscular function, high intensity interval training, kinematics, muscle strength, running, coordination variability

## Abstract

**Purpose:**

To examine the time course of recovery for gait and neuromuscular function immediately after and 24-h post interval training. In addition, this study compared the impact of different statistical approaches on detecting changes.

**Methods:**

Twenty (10F, 10M) healthy, recreational club runners performed a high-intensity interval training (HIIT) session consisting of six repetitions of 800 m. A 6-min medium intensity run was performed pre, post, and 24-h post HIIT to assess hip and knee kinematics and coordination variability. Voluntary activation and twitch force of the quadriceps, along with maximum isometric force were examined pre, post, and 24-h post significance HIIT. The time course of changes were examined using two different statistical approaches: traditional null hypothesis significance tests and “real” changes using minimum detectable change.

**Results:**

Immediately following the run, there were significant (*P* < 0.05) increases in the hip frontal kinematics and coordination variability. The runners also experienced a loss of muscular strength and neuromuscular function immediately post HIIT (*P* < 0.05). Individual assessment, however, showed that not all runners experienced fatigue effects immediately post HIIT. Null hypothesis significance testing revealed a lack of recovery in hip frontal kinematics, coordination variability, muscle strength, and neuromuscular function at 24-h post, however, the use of minimum detectable change suggested that most runners had recovered.

**Conclusion:**

High intensity interval training resulted in altered running kinematics along with central and peripheral decrements in neuromuscular function. Most runners had recovered within 24-h, although a minority still exhibited signs of fatigue. The runners that were not able to recover prior to their run at 24-h were identified to be at an increased risk of running-related injury.

## Introduction

Recreational running has seen a second boom in the early 2000s throughout Europe and North America (Scheerder et al., 2015), contributing to the growing popularity of recreational club running, with middle aged runners aged 34 and 54 years old forming 43% of road race competitors (Running USA, 2020). To improve their performance, these runners often train up to six sessions per week (Enoksen et al., 2011; Zinner et al., 2018), typically performing a combination of medium intensity continuous runs and high-intensity interval training (HIIT) (Enoksen et al., 2011; Wen et al., 2019). This growth in popularity has also contributed to the rise in the incidence of running-related overuse injuries (RROI). Videbaek et al. (2015) found that recreational runners sustain 7.7 RROI per 1,000 h of running, while van Gent et al. (2007) reported an incident rate of 19.4% to 79.3% following an examination of incidence rates across prospective, cross sectional, retrospective, and randomised clinical trials. A retrospective survey (*N* = 1145) of middle-aged (47 ± 11 years) recreational runners revealed that 49.8% were injured of whom 94% continued running despite experiencing pain (Linton and Valentin, 2018). Reducing injury rates in this group of runners would, therefore, have a widespread impact, however, understanding the aetiology of RROI remains a challenge and requires extensive examination.

Bertelsen et al. (2017) recently proposed a framework to explain RROI. Within this framework, RROI occurs when the tissue-specific load capacity is exceeded. The tissue-specific load capacity is a dynamic entity, reflecting the ability of the musculoskeletal system to tolerate load without getting injured. It reduces within and recovers between training sessions. Whether a runner exceeds this capacity will depend on their initial status at the start of the training session, which is heavily influenced by their level of recovery from previous training and the tissue-specific cumulative load experienced during their run. This cumulative load during the run is the product of the load per stride, the distribution of the load over the tissue structures per stride, and the number of strides taken [see Bertelsen et al. (2017) for a detailed description]. The load per stride is the impact force experienced which the neuromuscular system must control and distribute across the musculoskeletal system. If the accumulation of these repeated impact forces exceeds the runner's ability to control or tolerate them, then fatigue, defined as the inability to maintain an expected power output, will occur (Gandevia, 2001; Enoka and Duchateau, 2016). Fatigue could affect the control of gait mechanics and /or the distribution of the load across the tissue structures, thereby increasing the RROI risk.

Avoiding RROI requires the application of the correct training load relative to the athlete's state of recovery. This requires an understanding of both the extent of fatigue experienced within, and the time course of recovery between, training sessions. Traditionally, fatigue within a session has been considered metabolic, due to either substrate depletion or metabolite accumulation (Enoka and Duchateau, 2016). However, as runners fatigue, changes in both running gait and neuromuscular function have been observed. Fatigue-induced changes in gait have been shown in the frontal plane, for example, hip adduction angle. These changes in gait with fatigue are usually detectable after extreme fatigue, or more likely exhaustive exercise e.g., after a prolonged run to exhaustion or race (Nicol et al., 1991b; Millet et al., 2002, 2003; Place et al., 2004; Dierks et al., 2008; Bazett-Jones et al., 2013). Runners seldom undertake such exhaustive events, most of their running consists of training sessions, which although sometimes hard, are seldom to exhaustion. Training frequency far outweighs that of competing, meaning runners are more likely to sustain a RROI within training. Despite this, there has been a limited examination of the fatigue experienced during typical training sessions. Riazati et al. (2020) examined the effect of medium intensity continuous runs and HIIT on gait and muscular strength. They reported gait and strength decrements following both training types, with HIIT inducing greater changes. Compared to healthy runners, injured runners with patellofemoral pain syndrome (PFPS) or iliotibial band syndrome (ITBS) show greater hip frontal plane movement (Noehren et al., 2007, 2014; Dierks et al., 2008; Powers, 2010). The gait of these injured runners is similar to that seen in healthy runners as they fatigue, which would support the hypothesis that fatigue-induced changes in gait increase the risk of RROI.

Changes in gait with fatigue, are not just limited to joint angles and ranges of motion, there are also changes in movement coordination and variability (Chen et al., 2020). Increases in variability have been associated with a reduced ability to tolerate force absorption following ground contact (Mizrahi et al., 2000), potentially increasing injury risk (Baida et al., 2018). Furthermore, changes in variability could reflect a loss of movement control (Nordin et al., 2017). A reduction in maximal knee extensor (KE) isometric strength found during the last 5-km of a 20-km time trial, was highly correlated (*r* = *0.70*) with voluntary activation of the KE measured by twitch interpolation (Ross et al., 2010). The aetiology of changes in gait kinematics, coordination variability, load tolerance, and muscle force production is not fully resolved, but likely has both central and peripheral components i.e., proximal and distal to the neuromuscular junction. Following marathon and ultra-marathon races, both central and peripheral mechanisms of fatigue are evident; subsequent recovery can take several days. As previously noted, these are extreme events and do not reflect regular training. The extent and time-course of recovery for both central and peripheral mechanisms following typical training sessions warrant further investigation. Failure to recover from a previous training session would result in a decrease in the specific load capacity within Bertelsen's model, thereby increasing the risk of developing an RROI.

Traditionally, exercise scientists have examined changes in gait, whether due to fatigue or some other intervention, using null-hypothesis statistical tests (NHST). Considerable inter-individual variation in fatigue response was found post marathon (Nicol et al., 1991a), with runners showing changes in gait both above and below pre-marathon values. Null-hypothesis statistical testing uses grouped data to identify an overall response; the nuances of the inter-individual responses are thereby overlooked. Differentiating real change from random variation is problematic when trying to identify individual responses. Minimum detectable change (MDC) offers a potential solution; it is a confidence interval approach based upon test–retest reliability. Where an individual changes by more than MDC, this would represent a real change. Recently, Riazati et al. (2020) and Bramah et al. (2021) used this approach with the former reporting different findings when using NHST and MDC approaches.

Fatigue, both multifactorial and transient, comprises of central and peripheral components. This study had dual aims, firstly to identify these multifactorial changes using gait kinematics, coordination variability, muscle force production, and muscle activation immediately post and 24-h after a typical high-intensity interval session in middle aged recreational club runners. Secondly, to compare and contrast the effect of using two different statistical approaches (NHST and MDC) to detect these changes.

## Methods

### Research Design

A time-series design was used to observe changes in neuromuscular function, force production, kinematics, and running coordination variability pre, post, and 24-h post a HIIT session. It was neither feasible nor appropriate, to perform a HIIT session on consecutive days. Recreational masters age group club runners tend to run at a range of different intensities to improve performance and are likely to perform a medium intensity continuous run the day following a high intensity session (Zinner et al., 2018). A standard pace run (SPR) was, therefore, used pre, immediately post, and 24-h post HIIT to examine changes in kinematics and running coordination variability at a common speed.

### Participants

Following an *a priori* power analysis based on the kinematic variables in Riazati et al. (2020) (α = 0.5 and β = 0.20; desired effect size of 0.66) and subsequent institutional ethical approval, 20 healthy, experienced (running for at least 2 years), recreational masters age group club distance runners (N = 10 male; N = 10 female) were recruited (see Table 1). All the runners trained regularly, participating in HIIT, or similar type training session, at least once a week, most weeks, at their running club. All runners in this study performed between one to two HIIT sessions per week and also completed races ranging from 5 K to ultra-marathons. Table 1 shows participant characteristics, treadmill speeds, and interval duration. Participants were excluded if they had not competed in an organised race within the previous 2 years, were not part of an affiliated running club, or had experienced any type of lower extremity injury that prevented them from running for more than a week in the past 6 months. Further exclusion criteria included any cardiovascular or neurological conditions or an allergy to the adhesive material. Medical history was pre-screened via a self-reported questionnaire; eligible participants provided informed written consent prior to testing sessions.

**Table 1:** Descriptive characteristics of participants along with speeds, durations, $\overset{∙}{v}o_{2}$ max represented as mean ± standard deviation for both Hight-Intensity Interval Training session (HIIT) and standard pace run (SPR).

	**Female**	**Male**
	**(*n* = 10)**	**(*n* = 10)**
Age (years)	43.2 ± 4.5	43.0 ± 5.0
Height (cm)	165.5 ± 6.4	176.5 ± 7.8
Mass (kg)	61.4 ± 11.4	78.3 ± 9.3
HIIT Speed (m.s^−1^)	3.9 ± 0.4	4.3 ± 0.5
HIIT rep duration (min:sec)	03:24 ± 20(s)	03:10 ± 22(s)
SPR pace (m.s^−1^)	3.1 ± 0.3	3.4 ± 0.4
$\overset{∙}{V}$O_2_ max (ml.kg^−1^.min^−1^)	52.5 ± 6.2	55.3 ± 5.0

### Procedure

**Figure 1:**
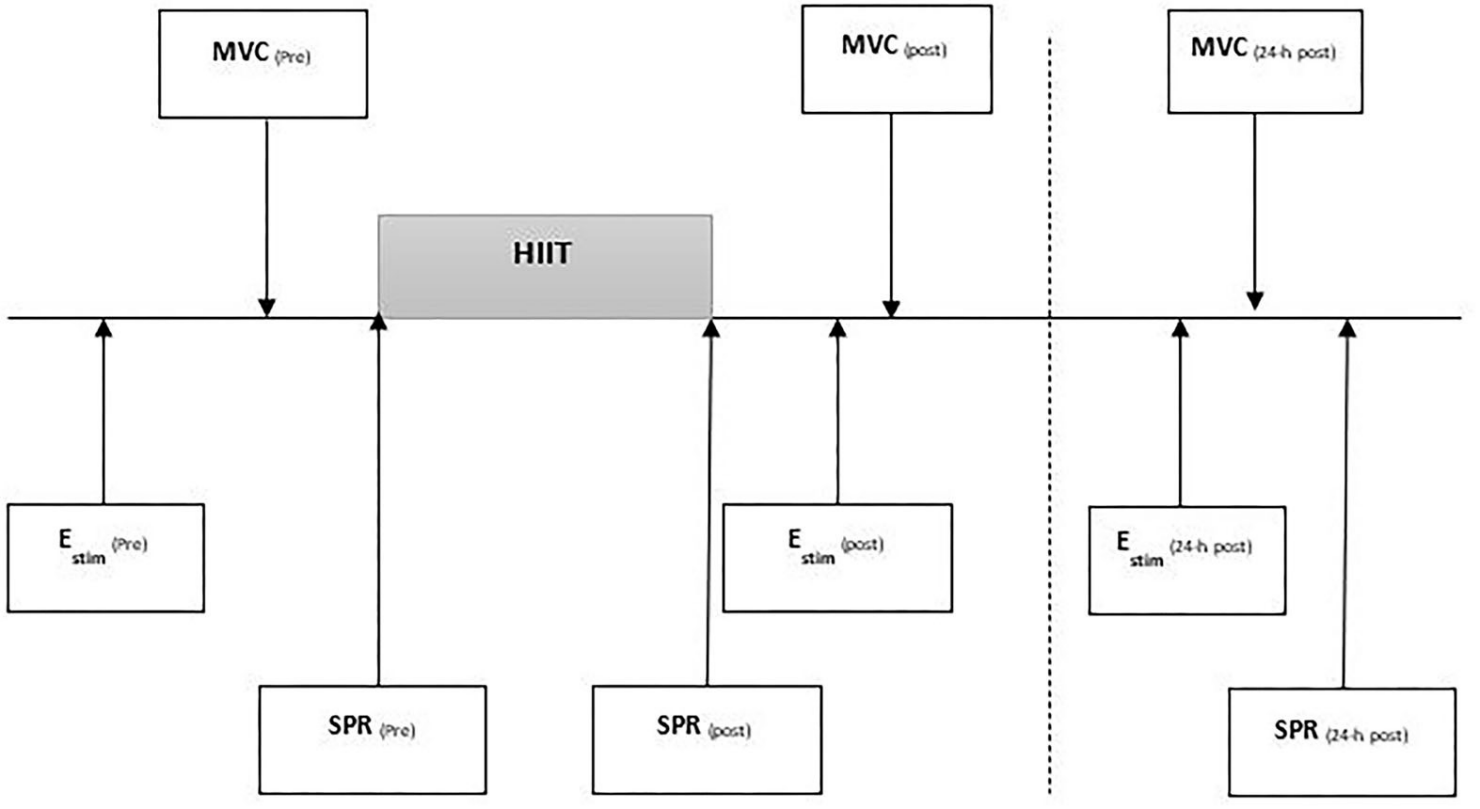
Testing procedure outlining timing of examination of the protocol containing electrical stimulation (E_stim_), maximal voluntary contraction (MVC) for hip and knee strength testing, standard pace run (SPR). All tests performed pre, post, 24-h post a high intensity interval training run (I-MT).

Each runner visited the laboratory three times (see Figure 1). Visit 1 was a preliminary session to determine the intensities for the HIIT and SPR and familiarise runners with hip strength and electrical stimulation (E_Stim_) procedures (see Figure 1). During visit 2, hip strength and E_Stim_ were measured pre and immediately-post HIIT. Gait kinematics were measured during SPR conducted before and immediately after the HIIT. During the final visit, 24-h post HIIT, runners again completed measures of hip muscular strength, the E_stim_ protocol, and SPR. All sessions were conducted at the same time of day to minimise diurnal variation (Reilly and Garrett, 1998). Runners were asked to wear the same footwear throughout and follow their habitual dietary regimen while refraining from high volume or intensity training within 48 h before testing They were also asked to refrain from any activity following the HIIT session prior to 24-h post assessment.

### Preliminary Testing

Initial measurements of mass, stature, and all kinanthropometric measures were taken according to ISAK guidelines, by an ISAK qualified practitioner. Runners completed an incremental treadmill (ELG2, Woodway, Germany) test to determine maximum steady state and $\overset{∙}{V}$O_2_ max. Expired gases were analysed using a Cortex Metalyser 3B (Leipzig, Germany), calibrated according to the manufacturer's instructions prior to each test. A 5-min warm-up run was completed prior to testing.

The sub-maximal test consisted of a series of incremental 4-min stages at a 0% gradient, separated by a 60-s recovery (Smith and Jones, 2001). Between stages, a fingertip capillary blood sample was taken for analysis of blood lactate concentration (Biosen C-line, EKF diagnostics, Germany). Running speed increased by 1 km·h^−1^ per stage until lactate turnpoint (LTP) was exceeded. LTP was defined as a second, steeper, more sustained, rise in blood lactate (Smith and Jones, 2001) and used to identify a maximum steady state. Following a 15-min recovery, participants completed a $\overset{∙}{V}$O_2_ max test, with the initial speed set at 4 km·h^−1^ below the speed at LTP, again 0% gradient was used throughout. The treadmill speed increased by 0.5 km.h^−1^ every 30 s until volitional exhaustion occurred. Breath by breath $\overset{∙}{V}$O_2_ data were 30-s averaged; the highest value was taken as $\overset{∙}{V}$O_2_ max (Billat et al., 2001; Midgley et al., 2006).

### Setting HIIT and Standard Paced Run Speeds

All runs were performed on a treadmill (ELG2, Woodway, Germany). Treadmill running has been shown to be comparable to overground running (Hooren et al., 2020), and offers a level of data capture not possible in a more, ecologically valid field-based setting. The duration and speed of both HIIT and SPR were individualised based on the individual's speed at $\overset{∙}{V}$O_2_ max (*s*$\overset{∙}{V}$O_2_ max) and LTP (*s*LTP). The HIIT session was a protocol previously shown to cause fatigue (James and Doust, 2000; Riazati et al., 2020), while all runners ran the same distance, the time taken to complete the HIIT was different (see Table 1). It consisted of six 800-m repetitions at 1 km·h^−1^ below *s*$\overset{∙}{V}$O_2_ max with a 1:1 work: rest ratio. The recovery was active, with participants walking at 4 km·h ^−1^. The SPR was halfway between the speed of lactate threshold, the first rise in blood lactate above baseline, and *s*LTP. The repetition speed was determined from a regression equation generated by plotting the averaged final minute $\overset{∙}{V}$O_2_ for each 4-min stage against RS from the sub-maximal test. This relationship was extrapolated up to $\overset{∙}{V}$O_2_ max to identify the speed at $\overset{∙}{V}$O_2_ max (s$\overset{∙}{V}$O_2_max). The speed of the repetitions was identified as 1 km·h^−1^ under s$\overset{∙}{V}$O_2_ max. replicating James and Doust (1998).

### Hip Muscular Strength

In order to examine muscle function and fatigue, maximum isometric force generating capability was examined (Gandevia, 2001) in the hip musculature pre, immediately-post, and 24-h post HIIT. A handheld dynamometer (Lafayette Instruments, IN, USA) was used to measure maximum voluntary isometric contraction (MVC) at the hip and knee. The handheld dynamometer was secured on the limb of the runner using a Velcro strap; furthermore, a non-elastic strap was placed around an examination table to remove tester strength bias. All hip testing positions were in accordance with Bazett-Jones et al. (2013) and included testing for hip abduction, adduction, extension, flexion, internal rotation, and external rotation, performed in respective order. Participants were asked to perform one set of three maximal effort trials for each movement in a 5-s ramp protocol, exerting maximum force against the dynamometer during the final 3 s. The highest value was recorded. There was 30 s of rest between each effort and a 1-2 min of rest between each muscle group (Bazett-Jones et al., 2013). The strength measures were recorded in Newtons and normalised to body weight (Bazett-Jones et al., 2013; Riazati et al., 2020). Measurements of lateral epicondyle to greater trochanter were used for hip strength measure normalisation.

### Neuromuscular Function Assessment

Electrical stimulation of the femoral nerve was used to assess the contribution of the central and peripheral mechanisms towards neuromuscular fatigue and recovery of the quadriceps (Brownstein et al., 2017). These measures were taken pre-SPR, immediately post (within one minute of completing the HIIT), and 24-h post HIIT.

A calibrated load cell (RDP load cell model RLT; Wolverhampton, UK) was attached to a fixed custom-built isometric force chair and attached to the runner's leg at the superior malleoli via a noncompliant cuff. The load cell was used to record muscle force (N) during maximum voluntary contraction (MVC) of the knee extensors. The isometric force chair was situated near the treadmill to ensure a rapid examination of neuromuscular function following the HIIT session. Immediately following the HIIT session, runners dismounted the treadmill, and hip markers were removed to connect electrodes for the motor nerve stimulation. Participants were seated in the custom-built chair with their hips and knees flexed to 90°; this process took no longer than 30 s.

To examine central drive, voluntary activation (VA) via motor nerve stimulation and potentiated quadriceps twitch force (Q_tw_,_pot_) were examined. In addition, the amplitude of the Q_tw_,_pot_ can be used to measure the contractile function of the muscle, providing an added measure for the peripheral drive (Brownstein et al., 2017). Muscle fatigue was observed from a drop in force production from the maximal voluntary contraction of the KE, along with the isometric strength assessments of the hip musculature previously explained.

Single and paired transcutaneous electrical muscle stimulation (100 Hz) was delivered to the right knee extensor via a constant-current stimulator (DS7AH, Digitimer Ltd., Hertforshire, UK). Circular 5-cm self-adhesive surface electrodes (model number, Nidd Valley Medial Ltd., North Yorkshire, UK) were used and the position of the electrodes was placed over the nerve in the femoral triangle, with the anode placed between the greater trochanter and the iliac crest (Weavil et al., 2015). The electrodes were also marked with indelible ink to ensure repeatable, consistent placement for post and 24-h post measurements. Electrical stimulation was delivered at rest to the relaxed muscle, beginning at 20 mA and increased incrementally, in a stepwise fashion by 20 mA, until a plateau occurred in quadriceps twitch amplitude (Q_tw_, N). This was performed only once on each visit; right at the start. The plateau intensity was then increased by 30% to ensure supramaximal stimulation. Subsequently, runners completed six isometric MVCs of the knee extensors with a duration of between 3 and 5 s, separated by 60 s of rest. During the final three MVCs, paired electrical stimuli (100 Hz) were delivered during, and 2-s post, contraction to assess VA. Single pulse electrical stimuli were delivered 5-s post MVC to assess quadriceps potentiated twitch force (Q_tw_,_pot_). Voluntary activation was quantified by comparing the superimposed twitch force (SIT) during MVC with the amplitude of the Q_tw_,_pot_ elicited 2-s after MVC at rest (Goodall et al., 2014; Brownstein et al., 2017). Voluntary activation percentage was expressed as:


(1) $$\begin{array}{r} VA\;\left(\%\right)=\left[\;1-\left(\frac{SIT}{Q_{tw,pot}}\right)x\;100\right] \end{array}$$

In our laboratory, the interclass correlation (ICC) scores for neuromuscular function are r = 0.98, 0.86, and 0.88 for MVC, VA, and Q_tw, pot_, respectively (Brownstein, 2018).

### Motion Analysis

Running mechanics were captured during each SPR using a 14-camera 3-dimensional motion analysis system (T-120/T-140; Vicon Motion Systems Ltd., Oxford, England) sampling at 250 Hz and calibrated before each session. The data were recorded using a lower body Plug-in Gait model following the procedure of Riazati et al. (2019, 2020). Retroreflective markers were placed bilaterally on the anterior superior iliac spine, posterior superior iliac spine, thigh, lateral epicondyles of the femur, lateral shank, lateral malleoli, base of the 2nd metatarsal, and calcaneus. The markers were carefully placed by the same researcher throughout, the desired markers were secured with double sided tape and the surrounding base was also taped down with double sided tape. Wand markers were used for the thigh and shank to obtain rotational movements of the joints. Runners wore compression leggings, with holes cut to allow markers to remain visible, to ensure the markers remained in place. The hip markers were taped around the hip, to ensure they remained in place throughout the run, using soft adhesive tape to avoid impeding hip movement. Static capture was performed 3 s prior to treadmill running sessions. This was processed using the static Plug-in Gait model and static subject calibration. During the SPR, motion analysis of kinematics and coordination variability data were recorded for 25-s at the end of the first minute.

### Data Analysis

All markers were labelled and marker trajectories were filtered using a fourth order low-pass Butterworth Filer via a dynamic plug-in gait model with a 6 Hz cut-off frequency, within the motion analysis software (Nexus 2.0, Vicon Motion Systems Ltd, Oxford, England). Gait identification was achieved through visual inspection of foot strike and toe off for 20 consecutive strides (Riazati et al., 2019). Maximum angle (max) and range of motion (RoM) of the hip and knee in the sagittal and frontal planes, during the stance phase, were extracted. All processed motion analysis data were analysed using a custom script in Matlab (2018a, The Mathworks, Inc. Natick, MA, USA).

### Coordination Variability

Coordination variability of interactions between sagittal (flexion/extension) and frontal (abduction/adduction) planes of motion for the hip and knee joint couplings were analysed using continuous relative phase variability (CRPV) and coupling angle variability (CAV) through vector coding. The selection of these two applications of dynamical system theory for variability was based on Miller et al. (2010), who found that both were valid methods for examining running variability. All variability analyses were processed using a custom script in Matlab (2018a, the Mathworks, Inc, Natick, MA, USA).

For both CRPV and CAV, interactions of the hip and knee joints were examined during treadmill running from 20 consecutive stance phase cycles. For full details of the procedures see Riazati et al. (2020).

### Statistical Analysis

To examine the effects of the HIIT immediately and 24-hr following, this study used two statistical approaches: null hypothesis testing for group examination and minimum detectable change for individual assessment.

#### Null Hypothesis Testing

All data were checked for normality using Q-Q plots and deemed normally distributed. Mean and standard deviation was calculated for all variables. A one-way repeated measures ANOVA was used to assess changes over time for kinematics and joint coupling variability during SPR in addition to strength and neuromuscular function. Where appropriate Tukey's LSD test was used *post hoc*. Data were tested for sphericity using Mauchly's test. Effect sizes were calculated according to Cohen (2013) and interpreted as small (≥ 0.2), moderate (≥ 0.5), and large (≥ 0.8). The level of significance was set at *P* < 0.05. All statistical analyses were performed in SPSS v22.0 (SPSS Inc., Chicago, IL, USA).

#### Minimum Detectable Change

Minimum detectable change was calculated using the method of Weir (2005). An individual was considered to have fatigued when their scores immediately post or 24 h post HIIT, had changed by more than, or equal to, MDC set at a 95% confidence interval. The MDC were derived from our own reliability data, the tables are attached to the Supplementary Files 1–4 (SDC 1-4, SEM, and MDC values for measures of muscular strength, kinematics, and variability).

## Results

### Muscular Strength

#### Null Hypothesis Testing

All muscular strength measures decreased significantly with time following the HIIT (see Table 2) showing small to moderate effect sizes. Hip internal rotators declined over time (F_2, 19_ = 9.50, *P* = 0.001) with a 10.8% reduction in strength post HIIT, remaining unchanged at 24-h post (10.4%), *post hoc* analysis revealed a significant reduction both post (*P* < 0.001, d = 0.39) and 24-h post (*P* = 0.009, d = 0.42). In hip abduction strength, there was significant reduction with time (F_2, 19_ = 34.14, *P* < 0.001). Runners exhibited an 11.2% reduction in hip abduction strength post HIIT (*P* < 0.001, d = 0.41) with minimal recovery the following day (9.5%), (*P* < 0.001, d = 0.41). Hip adduction strength was reduced significantly with time (F_2, 19_ = 18.5, *P* < 0.001), at post (*P* < 0.001, d = 0.37) and 24-h post (*P* = 0.002, d = 0. 26). A significant reduction was found in hip external rotator strength with time (F_2, 19_ = 9.54, *P* < 0.001), there was a 12.8% reduction post HIIT (*P* < 0.001, d = 0.47), however this decline (5.8%) was no longer significant at 24-h post (*P* = 0.267, d = 0.23). Likewise, hip flexion strength showed a decline with time (F_2, 19_ = 25.94, *P* < 0.001), while *post hoc* analysis showed significant reduction post HIIT (11.3%) (*P* < 0.001, d = 0.39) but not at 24-h post (9.1%) (*P* = 0.051, d = 0.34).

**Table 2:** Hip strength measures, body mass-normalised (kg.kg^−1^) represented as mean ± standard deviation for pre, post, and 24-h training run along with effect size (ES; Cohen's *d*) and individuals exceeding minimum detectable change (MDC) for each variable at post and 24-h post compared to pre.

	**Mean** **±SD**					
	**(ES)**					
**Strength measures**	**Pre**	**Post**	**24-h**	**Time**	**Pre vs. post MDC**	**Pre vs. 24-h MDC**
Hip abduction	0.500 ± 0.16	0.444 ± 0.15*** (d = 0.36)	0.452 ± 0.15*** (d = 0.30)	†	2	3
Hip adduction	0.408 ± 0.40	0.277 ± 0.10*** (d = 0.45)	0.289 ± 0.09* (d = 0.41)		0	0
Hip flexion	0.438 ± 0.13	0.388 ± 0.12*** (d = 0.39)	0.398 ± 0.14 (d = 0.29)	†	0	0
Hip internal Rotation	0.195 ± 0.06	0.174 ± 0.06*** (d = 0.35)	0.176 ± 0.05* (d = 0.34)	†	3	4
Hip External Rotation	0.219 ± 0.06	0.191 ± 0.05*** (d = 0.51)	0.208 ± 0.06 (d = 0.18)	†	2	2

*Effect size in comparison with baseline*.

^†^denotes significant effect with time. Significant differences in comparison with baseline indicated by ^*^P < 0.05, ^***^P < 0.001.

#### Minimum Detectable Change

The use of MDC showed that two runners had reduced hip abduction strength after HIIT beyond MDC, while three runners experienced a reduction beyond MDC at 24-h post. In hip internal rotation, three runners exceeded MDC immediately post HIIT but all had recovered by 24-h post. Post HIIT, two runners experienced a reduction in hip external rotation above MDC. Of these, one runner experienced a further drop at the 24-h post, while the other had recovered. For force measures of hip adduction and hip flexion, no runner reduced force below MDC at any time point.

### Neuromuscular Function

#### Null Hypothesis Testing

Runners showed decrements within all neuromuscular function measures (see Table 3). Knee extensor MVC showed a significant reduction in force production over time (F_2, 13_ = 19.74, *P* < 0.001). Force production declined by 8.1% (*P* < 0.001, d = 1.21) pre to immediately post HIIT and remained 3.2% lower 24 h -post (*P* = 0.022, d = 0.15). Similarly, Q_tw_,_pot_ exhibited a significant reduction over time (F_2, 14_ = 4.08, *P* = 0.017). Quadriceps potentiated twitch force was reduced from pre to immediately post HIIT (*P* = 0.013, *d* = 0.56), but had recovered by 24-h post (*P* = 0.393, *d* = 0.12). There was a significant change in VA over time (F_2, 14_ = 17.25, *P* < 0.001) suggesting runners were affected by central fatigue. Voluntary activation dropped 7.3% from pre to immediately post (*P* < 0.001, *d* = 1.07), recovering to a 2.0% deficit (*P* = 0.013, *d* = 0.37).

**Table 3:** Neuromuscular function measures of maximum voluntary contraction (MVC) of the knee extensors, voluntary activation percentage (VA%), and quadriceps resting twitch potential (Q_tw_,_pot_, ,N) at pre, post, and 24-h of training run represented as mean ± standard deviation along with effect size (ES; Cohen's *d*) and individuals exceeding minimum detectable change (MDC) for each variable at post and 24-h post compared to pre.

	**Mean** **±SD**					
	**(ES)**					
	**Pre**	**Post**	**24-h**	**Time**	**Pre vs. post MDC**	**Pre vs. 24-h MDC**
MVC (N)	487.4 ± 31.8	448.7 ± 31.9*** (d = 1.21)	471.8 ± 141.9* (d = 0.15)	†	6	5
Q_tw_,_pot_ (N)	188.6 ± 43.9	162.9 ± 48.5* (d = 0.56)	183.1 ± 49.3 (d = 0.11)	†	8	2
VA%	93.2 ± 4.4	86.4 ± 7.8*** (d = 1.07)	91.3 ± 5.8* (d = 0.37)	†	15	4

*Effect size in comparison with baseline*.

^†^denotes significant effect with time. Significant differences in comparison with baseline indicated by ^*^P < 0.05 and ^***^P < 0.001.

#### Minimum Detectable Change

Analysis of individual KE MVC scores revealed that six runners had reduced force production beyond MDC immediately post HIIT, with only one runner recovering at 24-h post. For Q_tw_,_pot_ immediately post HIIT, eight runners exhibited a reduction larger than MDC, with two of them failing to recover 24-h later. Furthermore, VA% dropped by more than MDC for 15 runners, with four runners still remaining impaired at 24-h post. Two runners exceeded MDC for all neuromuscular function measures at both post and 24-h post.

### Kinematics

#### Null Hypothesis Testing

There was a significant increase in both hip maximum angle (F_2, 19_ = 11.05, *P* = 0.001) and RoM (F_2, 19_ = 17.39, *P* < 0.001) in the frontal plane (see Table 4). For maximum angle, *post hoc* analysis revealed a large increase in hip adduction both immediately post (*P* < 0.001, *d* = 0.91) and at 24-h post (*P* < 0.001, *d* = 0.86). Similarly, for RoM angle, there was a large increase in hip adduction during SPR immediately-post (*P* < 0.001, *d* = 0.85) which, although slightly reduced, remained elevated at 24-h post (*P* < 0.001, *d* = 0.74).

**Table 4:** Hip and knee joint kinematics of maximal angles and range of motion (RoM) angles in the sagittal and frontal plane movements of standard pace run represented as mean ± standard deviation for pre, post, and 24-h post training run along with effect size (ES; Cohen's *d*) and individuals exceeding minimum detectable change (MDC) for each variable at post and 24-h post compared to pre.

		**Mean** **±SD**					
		**(ES)**					
		**Pre**	**Post**	**24-h**	**Time**	**Pre vs. post MDC**	**Pre vs. 24-h MDC**
Maximum angles (deg)							
	Hip sagittal	38.8 ± 6.2	38.2 ± 7.2 (d = 0.09)	37.4°± 5.8 (d = 0.23)		2	3
	Hip frontal	9.6 ± 3.9	13.3 ± 4.2*** (d = 0.91)	12.7°± 3.3*** (d = 0.86)	†	11	9
	Knee sagittal	42.9 ± 14.7	42.9 ± 6.5 (d = 0.26)	43.7°± 4.7 (d = 0.20)		1	4
	Knee frontal	0.3 ± 4.3	−0.1 ± 3.3 (d = 0.08)	2.2°± 4.0 (d = 0.46)	†	0	1
ROM angles (deg)							
	Hip sagittal	43.5 ± 6.5	44.6 ± 5.5 (d = 0.18)	43.8°± 4.8 (d = 0.05)		3	1
	Hip frontal	11.9 ± 3.2	14.9 ± 3.8*** (d = 0.85)	14.5°± 3.8*** (d = 0.74)	†	7	3
	Knee sagittal	31.3 ± 13.4	27.6 ± 4.3 (d = 0.38)	29.5°± 5.1 (d = 0.17)	†	4	6
	Knee frontal	5.5 ± 2.6	4.8 ± 1.7 (d = 0.32)	5.3°± 2.7 (d = 0.08)		1	1

*Sagittal plane positive values represent flexion; negative values represent extension*.

*Frontal plane positive values represent adduction angles; negative value represent abduction movement*.

*Effect size is comparison with baseline*.

^†^denotes significant effect with time. Significant differences in comparison with baseline indicated by ^***^P < 0.001.

Knee kinematics showed a main effect for time in sagittal plane RoM (F_2, 19_ = 5.32, *P* = 0.015, *d* = 0.38) and frontal plane maximum angle (F_2, 19_ = 3.65, *P* = 0.046, *d* = 0.74). *Post hoc* analysis failed to show a significant change either immediately post, or at 24-h post, compared to baseline for either variable.

#### Minimum Detectable Change

Individually, 11 runners exhibited fatigue effects with an increase in hip frontal plane maximum angle post HIIT. Twenty-four hours later, nine runners were still showing signs of fatigue; more than any other kinematic variable. Seven runners experienced an increase above MDC for hip frontal plane RoM immediately post HIIT, with three still exceeding MDC 24-h post. Two runners experienced changes beyond MDC for hip sagittal plane maximum angle immediately post HIIT; one higher, the other lower. However, 24-h later, the runner with elevated sagittal plane movement had recovered, while the runner with decreased sagittal plane movement experienced a further drop.

At the knee, one runner decreased knee sagittal plane maximum angle beyond MDC post HIIT. By 24-h post, four runners experienced a change beyond MDC; for one runner it was a reduced angle, and the remaining three experienced increased knee flexion. Four runners displayed a reduced sagittal plane knee RoM immediately post HIIT that exceeded MDC. However, at the 24-h post, six runners exceeded MDC, with two changing from a decreased to increased RoM above MDC, while the others maintained their decreased RoM. No runner experienced a change above MDC for maximum angle of the knee in the frontal plane immediately post HIIT, and only one runner changed above MDC at 24-h post. This runner altered their gait strategy from running in abduction immediately post HIIT to adduction at 24-h post. For knee RoM angle, only one runner exceeded MDC immediately post HIIT with an increased RoM angle that was still present 24-h later.

### Coordination Variability

#### Null Hypothesis Testing

Running coordination variability, assessed by vector coding coupling angle, revealed no significant change with time (see Table 5). Continuous relative phase variability revealed a significant change (*P* < 0.05) with time for all coupling interactions. *Post hoc* analysis showed that the increased variability observed, both immediately post and 24-h post HIIT, were significant for all examined interactions (Table 4).

**Table 5:** Joint coupling interactions of sagittal and frontal plane movements of the hip and knee measured through vector coding coupling angle variability (CAV) and continuous relative phase variability (CRPV) during SPR runs at pre, post, and 24-h post training run represented as mean ± standard deviation along with effect size (ES; Cohen's *d*) and minimum detectable change (MDC) for each variable at post and 24-h post compared to pre.

		**Mean** **±SD**					
		**(ES)**					
		**Pre**	**Post**	**24-h**	**Time**	**Pre vs. post MDC**	**Pre vs. 24-h MDC**
CAV (deg)								
	Hip_flex/ext_- Knee_flex/ext_	66.7 ± 5.3	67.3 ± 4.9 (d = 0.12)	66.2 ± 4.5 (d = 0.10)		2	2
	Hip_flex/ext_-Knee_abd/add_	66.8 ± 6.3	67.3 ± 5.2 (d = 0.09)	68.0 ± 4.6 (d = 0.22)		16	15
	Hip_abd/addt_-Knee_flex/ext_	71.7 ± 1.8	71.4 ± 2.5 (d = 0.14)	71.6 ± 2.2 (d = 0.05)		7	7
	Hip_abd/addt_-Knee_abd/add_	68.4 ± 4.8	68.6 ± 4.2 (d = 0.44)	69.8 ± 3.3 (d = 0.34)		14	8
CRPV (deg)								
	Hip_flex/ext_- Knee_flex/ext_	12.9 ± 3.9	17.8 ± 10.1* (d = 0.64)	19.7 ± 13.0* (d = 0.74)	†	3	6
	Hip_flex/ext_-Knee_abd/add_	9.1 ± 4.8	15.2 ± 14.1* (d = 0.56)	20.3 ± 16.5* (d = 0.90)	†	3	7
	Hip_abd/addt_-Knee_flex/ext_	16.2 ± 4.1	23.5 ± 13.9* (d = 0.73)	26.0 ± 12.6** (d = 1.05)	†	5	4
	Hip_abd/addt_-Knee_abd/add_	11.0 ± 4.9	21.3 ± 14.3** (d = 0.93)	23.3 ± 14.7** (d = 1.10)	†	6	9

*Effect size is comparison with baseline*.

^†^denotes significant effect with time. Significant differences in comparison with baseline indicated by ^*^P < 0.05, ^**^P < 0.01.

#### Minimum Detectable Change

For CAV, the individual assessment showed that in Hip_flex/ext−_Knee_abd/add_ 16 runners exhibited a change above MDC immediately post HIIT. Of those 16 runners, seven had increased variability immediately post HIIT, changing to decreased variability 24-h post. The remaining nine runners exhibited decreased variability immediately post; at 24-h post, one had recovered, the rest remained fatigued. Two runners exhibited an increase in variability above MDC both immediate post and 24-h post for Hip_flex/ext−_Knee_flex/ext_ coupling. Seven runners experienced an increase above MDC change for coupling of Hip_abd/add_-Knee_flex/ext_ immediately post and 24-h post; three showed increased variability immediately post and two failed to recover at 24-h post. Immediately post HIIT, 14 runners increased variability above MDC in Hip_abd/add_-Knee_abd/add_ coupling, eight of whom failed to recover at 24-h post.

Individual assessment of Hip_flex/ext−_Knee_flex/ext_ showed that three runners experienced a change above MDC immediately post and while the three remained increased, an additional three runners exhibited increased variability at 24-h post. In Hip_flex/ext−_Knee_abd/add_, three runners increased CAV above MDC both immediately post HIIT and 24-h post. Four further runners showed increased variability 24-h post. For Hip_abd/add_-Knee_abd/add_, five runners exceeded MDC immediately post; all but one still exceeded MDC 24-h later. For Hip_abd/add_-Knee_flex/ext_ coupling, five runners exceeded MDC immediately post, and four remained elevated 24-h post. In all CRPV interactions, the runners had increased variability apart from one runner who reduced variability in all couplings except Hip_abd/add_ – Knee_flex/ext_.

## Discussion

This study found reduced hip and knee strength, increased frontal plane hip kinematics, and increased coordination variability following a HIIT session. In addition, this study is the first to not only report impairments to central and peripheral drive following a HIIT session but also that not all runners had recovered 24 h later. Null hypothesis significance testing showed fatigue-induced changes at a group level. The minimal detectable change was used to identify “real changes” at an individual level, revealing some conflicting, but mostly more variable and nuanced, findings.

### Gait and Hip Strength

The HIIT induced a statistically significant decline in strength for all hip strength tests immediately post, while inducing changes to hip frontal kinematics. Additionally, runners remained in a state of reduced strength (*P* < 0.05) the following day for hip abduction, adduction, and internal rotation. Similarly, hip frontal plane kinematics were also increased at 24-h. When analysing the data using MDC rather than NHST, a far more nuanced picture emerged, with very few runners having a drop in force production. Furthermore, not all runners experienced gait changes.

Individually, 11 runners showed fatigue-induced increases in maximum hip adduction angle immediately post HIIT, evidenced by exceeded MDC. Of these 11 runners, nine failed to recover, still exhibiting an increased hip adduction angle 24-h later. Furthermore, two of the 11 runners also exhibited a loss of strength in their hip abductors beyond MDC post HIIT. At 24-h post, three runners exceeded MDC in hip abduction strength; they also showed increased maximum hip frontal angle and RoM.

These findings are also supported by previous studies examining hip abductor strength pre and post prolonged runs (Dierks et al., 2008; Bazett-Jones et al., 2013; Riazati et al., 2020), where hip abductor strength was lower following a run along with increased hip adduction angle. The findings of this study further support the suggestion of Riazati et al. (2019) that the identification of potential risk factors for developing running related overuse injuries is better performed on an individual basis.

The causality of these alterations in running gait (e.g., the observed increase in hip adduction angle), has been mostly attributed to muscular decrements (Dierks et al., 2008; Noehren et al., 2014). Runners suffering from patellofemoral pain and iliotibial band syndrome exhibit dysfunction at the gluteus medius muscle, which can result in poor frontal plane movement control (Semciw et al., 2016). While muscle activity was not measured, a reduction in gluteus medius force production observed through decreased hip abduction strength is the likely cause for the increased hip adduction angles. The inability to control hip frontal movement can also contribute to increased strain on the IT band and stress on the patellofemoral joint (Noehren et al., 2007; Dierks et al., 2010; Powers, 2010). The findings of this study provide further support to the previous body of evidence suggesting reduced force production to be the cause of altered frontal kinematics (Dierks et al., 2010; Powers, 2010; Brown et al., 2016; Willwacher et al., 2020).

### Neuromuscular Function

The HIIT session induced decrements to both central and peripheral neuromuscular function immediately post, with reductions of 6.8% in VA, 8.1% in MVC, and 14% in Q_tw_,_pot_. Post 24-h, the runners remained in an impaired state (*P* <0.05) despite recovering to a reduction of 2.0% in VA, 3.2% in MVC, and 3.0% in Q_tw_,_pot_. The impairments immediately post were lower than those previously found. Following maximal repeated sprints Goodall et al. (2014) reported a drop of 12% in KE MVC and 23% in Q_tw_,_pot_. Similarly, Ross et al. (2010) observed a 15% in KE MVC and a 13% drop in VA following a 20-km run. The reduction in Q_tw_,_pot_, however, is slightly higher than runs of 1 h (13%) or 30 km (8%) reported by Davies and White (1982) and Millet et al. (2003), respectively. The decrement of VA in this study was lower than the previously reported reductions following an ultramarathon (13%), 24-h treadmill running (33%), and repeated sprints (9%) (Millet et al., 2002; Martin et al., 2010; Goodall et al., 2014). This observation, as a group, was however not unexpected as the mechanisms and extent of fatigue are exercise domain dependent. Fatigue resulting from prolonged activity in the moderate domain has been shown to be mostly of central origin (Burnley and Jones, 2016) while the HIIT was in the severe domain, inducing both central and peripheral decrements.

This study is the first to report individual impairments in neuromuscular function immediately and 24-h post HIIT. Sixteen of the 20 participants showed decrements in neuromuscular function, with four showing a loss of performance in all three measures. Peripheral fatigue was evident with decrements in the contractile function of the knee extensors. There was an impaired force output, measured through Q_tw_,_pot_, immediately post HIIT that persisted for 24-h (*P* < 0.05). Such a decrement in force output could be due to both metabolic and/or mechanical factors that influence excitation-contraction coupling along with action potential transmission at the sarcolemma (Allen et al., 2008). The reduced Q_tw, pot_ is an indicator of a reduction in the excitation-contraction coupling process. This could be at the cross-bridge level resulting from metabolic and mechanical disturbances, as well as impairments to neuromuscular transmission at the sarcolemma (Allen et al., 2008; Goodall et al., 2014). Moreover, it could be due to mechanical stresses e.g., a disorganisation of sarcomeres and Ca^2+^ handling interference (Skurvydas et al., 2016).

Central fatigue was evident immediately following HIIT with a large drop (ES = 1.06) in VA (*P* < 0.05) that was experienced by 15 participants, four of whom had failed to recover 24-h later. It is not possible to identify the supraspinal mechanisms that resulted in the central fatigue in this study, only that there was fatigue “upstream” of the peripheral nerve. A reduction in central drive while decreasing motor unit recruitment, from either firing frequency and/ or the number of motor units recruited, contributed to the decline in MVC (Gandevia, 2001). The HIIT session was performed in the severe domain, resulting in the accumulation of metabolites in the extracellular fluid e.g., H^+^, Pi, K^+^ which can stimulate type III and IV afferents reducing central drive (Amann, 2011). Central factors might therefore be responsible for some of the changes in coordination variability. Changes in gait variability could be seen as a “loss of control” rather than due solely to peripheral fatigue.

### Coordination Variability

By the end of the HIIT the runners were unable to maintain a stable level of coordination measured by CRPV (*P* < 0.5), this was still evident 24-h later. With CAV, there was no group effect, however, several runners exhibited an increase beyond MDC. Increased variability could be the result of decrements in force production and/or impairments in neuromuscular control having central and/or peripheral origins, as observed in this study. The detection of fatigue through variability could signal decrements in the contractile or neural function of the muscles when not examined directly. Each of the four runners who experienced an impairment to either central or peripheral drive at 24-h post concurrently had increased coordination variability in at least one coupling for either CAV or CRPV. The persistence of increased coordination variability at the 24-h post would suggest that the contractile and/or neural decrements might not have recovered. It is possible that if reduced variability can be used as a tool to discriminate between injured and non-injured runners (Seay et al., 2011), increased variability could provide a means to detect fatigue.

Schöner (1995) suggested that muscles and joints can be organised by the central nervous system to stabilise different task-specific performances. Instability can be identified as when motor systems, or processes that modulate coordinated movements, are unable to return to a certain state following small perturbations (Latash and Huang, 2015). Changes, both increases and decreases, in coordination variability by the end of the HIIT session show that two joint coordination instability had increased. These changes in coordination variability and gait (in)stability could be due to decrements in central nervous system function, with neurological patients exhibiting atypical, multi-joint coordination movement patterns. This can lead to compensatory changes in muscle activation strategies, for example an increased co-activation of agonist-antagonist muscle to impact gait deviation, thereby decreasing variability to improve stabilisation (Latash and Huang, 2015). By the end of the HIIT session, most runners were unable to maintain a stable running form. This was probably caused, in part, by impaired central drive, along with reduced capability of the gluteus medius muscle, working eccentrically, to act as a brake, resulting in increased variability.

Bartlett (2004) suggested that little or no variation in a movement would result in the same tissue being loaded at each ground contact, with potentially damaging consequences. Ferber and Pohl (2011) observed increased variability, albeit in walking, following locally induced fatigue in healthy participants. They attributed this to the diminished ability of the posterior tibialis to produce force, therefore requiring greater assistance from other muscles that contribute to the same joint movement in providing stability. Given the increase in hip abduction angle and reduction in hip abductor force production during the HIIT, the runners could have required a compensatory increase in the activation of other muscles contributing to this movement (e.g., gluteus minimus and tensor fascia lata) (Flack et al., 2014). Additionally, the decrease in muscle force could suggest a decreased recruitment which could explain the increase in hip adduction RoM. Increased coordination variability could, therefore, be considered as a mechanism to distribute impact loading as the muscle becomes fatigued. This requires further examination through direct measurement of muscle activity using electromyography. Furthermore, studies using wearable technology or a markerless motion capture system could enable this to be tested outside of a lab where more ecologically valid testing can be performed onlarge samples.

### Statistical Approaches

Two different statistical approaches were used within this paper: conventional null-hypothesis statistical tests and the detection of a “real change” using MDC. These approaches, at times, gave contrasting findings, while at other times showed agreement. Hip adduction strength is a good example, whereby a statistically significant reduction was seen immediately post HIIT (*P* < 0.001) which failed to recover by 24-h post (*P* < 0.05), whereas no runner showed reductions that exceeded the minimum detectable change. The approach adopted, therefore, dramatically altered the conclusion drawn. In this instance, in our opinion, the small to modest effect sizes support the conclusion drawn from using MDC. By contrast coordination variability of hip_flex/ext_ – knee_abd/add_, showed no significant difference at any point post HIIT, however this masked the considerable inter-individual responses. Immediately post-HIIT 16 of the 20 runners had changes in variability in excess of MDC, with seven increasing and nine decreasing, highlighting inter-individual variation. These inter-individual variations could explain the lack of statistical significance. After 24 h, all seven runners who had demonstrated increased variability, now showed a reduced variability (i.e., greater than MDC). Of the nine runners with reduced variability immediately post-HIIT, one recovered (i.e., fell back within MDC) and the rest continued to show reduced variability. Minimum detectable change offers the potential to detect more nuanced, individual strategies that might otherwise be overlooked.

### Implications

An increase in hip adduction angle has been considered to be a risk factor for the development of patellofemoral pain (Noehren et al., 2013). In this study, the MDC for hip adduction angle was 5.0°, beyond this we considered a runner to be at increased risk. Just over half of the runners in this study, exhibited a real change with an increase in hip adduction immediately post HIIT, only two of whom had recovered 24-later. This suggests that these runners could potentially start their next training session with an elevated risk of developing patellofemoral pain.

If runners recover from the fatigue, which most in this study did, then there is unlikely to be a risk of injury development. As runners often train on consecutive days, a lack of adequate recovery could lead to potential overuse injury development (Bertelsen et al., 2017). At 24-h post, all but four runners had recovered from impairments to both VA and Q_tw_,_pot_. This is not surprising as the vast majority of runners do not experience an injury after each training run, matching epidemiological data identifying 7.7 injuries every 1,000 h of running in recreational runners (Videbaek et al., 2015). This study highlights an appropriate method to identify the few runners who are at an increased risk of injury.

The use of MDC suggests increased injury risk is individual and that not only did each runner differ in risk at the end of the session but also with recovery pattern. The findings of this study further support the suggestion of Riazati et al. (2020), that assessing the potential risk of developing running related overuse injuries is better performed on an individual basis. Individual variations in the extent and origin of fatigue, along with recovery, further support the use of alternative statistical approaches to examine fatigue effects on runners.

## Conclusion

Following a HIIT session, middle-aged recreational club runners, experienced statistically significant impairments to both central and peripheral drive, along with changes in gait kinematics, gait variability, and a reduced ability to produce force at the hip and knee. Identifying individuals who changed by more than the minimum detectable change revealed considerable inter-individual variation and a more nuanced picture. The majority of runners showed a reduction in central drive and an increase in gait variability by the end of the HIIT session, reflecting a possible loss of motor control. Most of these runners were able to recover within 24-h, however, a small number failed to do so, and still had decrements in neuromuscular function, muscle force production, altered kinematics, and coordination variability. For these runners, running again before they have fully recovered could pose an increased risk of succumbing to an overuse injury.

## Data Availability Statement

The raw data supporting the conclusions of this article will be made available by the authors, without undue reservation.

## Ethics Statement

The studies involving human participants were reviewed and approved by Northumbria University. The patients/participants provided their written informed consent to participate in this study.

## Author Contributions

SR, PH, and NC contributed to the conception/design of the work along with interpretation of the data. MM contributed to the data analysis. All authors drafted the intellectual content and the final version.

## Conflict of Interest

The authors declare that the research was conducted in the absence of any commercial or financial relationships that could be construed as a potential conflict of interest.

## Publisher's Note

All claims expressed in this article are solely those of the authors and do not necessarily represent those of their affiliated organizations, or those of the publisher, the editors and the reviewers. Any product that may be evaluated in this article, or claim that may be made by its manufacturer, is not guaranteed or endorsed by the publisher.
