# Supplementary material for: Gait and Neuromuscular Changes Are Evident in Some Masters Club Level Runners 24-h After Interval Training Run
Source: Front Sports Act Living. 2022 Jun 2;4:830278. doi: 10.3389/fspor.2022.830278 (PMC9201250; doi:10.3389/fspor.2022.830278)
Supplement: Supplementary file 1 [file Table_1.DOCX]

| Supplementary Digital Content 1. Standard error of measurement (SEM) and minimum detectable change (MDC) values for strength measures of hip musculature. | | | | |  |  |
| --- | --- | --- | --- | --- | --- | --- |
|  |  |  | |  | |  |
|  | SEM | | MDC | |  |  |
|  |  | |  | |  |  |
| Hip Abduction | 0.031 | | 0.087 | |  |  |
| Hip Adduction | 0.031 | | 0.086 | |  |  |
| Hip Flexion | 0.035 | | 0.098 | |  |  |
| Hip Internal Rotation | 0.023 | | 0.064 | |  |  |
| Hip External Rotation | 0.014 | | 0.086 | |  |  |
|  | | | | |  |  |
