# Supplementary material for: Gait and Neuromuscular Changes Are Evident in Some Masters Club Level Runners 24-h After Interval Training Run
Source: Front Sports Act Living. 2022 Jun 2;4:830278. doi: 10.3389/fspor.2022.830278 (PMC9201250; doi:10.3389/fspor.2022.830278)
Supplement: Supplementary file 2 [file Table_2.DOCX]

| Supplementary Digital Content 2. Comparison Standard Error of Measurement (SEM), Minimum Detectable Changes (MDC), for maximum angles (Max) and range of motion (RoM) of stance phase in sagittal and frontal planes of motion for Knee, and Hip in both run-types. | | | | |  |
| --- | --- | --- | --- | --- | --- |
|  | SEM | MDC | SEM | MDC | |
|  | Sagittal (degrees) | | Frontal (degrees) | | |
| Knee Max | 2.0 | 5.5 | 2.7 | 7.5 | |
|  |  |  |  |  | |
| Knee RoM | 1.5 | 4.2 | 2.4 | 6.7 | |
|  |  |  |  |  | |
| Hip Max | 2.9 | 8.0 | 1.0 | 2.8 | |
|  |  |  |  |  | |
| Hip RoM | 1.4 | 3.9 | 2.0 | 5.5 | |
|  |  |  |  |  | |
