# Supplementary material for: Gait and Neuromuscular Changes Are Evident in Some Masters Club Level Runners 24-h After Interval Training Run
Source: Front Sports Act Living. 2022 Jun 2;4:830278. doi: 10.3389/fspor.2022.830278 (PMC9201250; doi:10.3389/fspor.2022.830278)
Supplement: Supplementary file 3 [file Table_3.DOCX]

| Supplemental Digital Content 3. Comparison of Intraclass Correlation Coefficients (ICC), Standard Error of Measurement (SEM), Minimum Detectable Changes (MDC), for Continuous Relative Phase Variability (CRPV) and Coupling Angle Variability (CAV). | | | | | | | | | | | | | | | | | | | |  |
| --- | --- | --- | --- | --- | --- | --- | --- | --- | --- | --- | --- | --- | --- | --- | --- | --- | --- | --- | --- | --- |
|  |  |  |  | |  | | |  | | |  |  | | |  | | |  | | |
|  |  |  | CRPV | | | | | | | |  | CAV | | | | | | | | |
|  |  |  | | SEM | | | MDC | | | |  | | | SEM | | MDC | | |  |  |
|  |  |  | |  | | |  | | | |  | | |  | |  | | |  |  |
| Hip_flex/ext_- Knee_flex/ext_ |  |  | | 3.8 | | | 10.5 | | | |  | | | 2.1 | | 5.8 | | |  |  |
|  |  |  | |  | | |  | | | |  | | |  | |  | | |  |  |
| Hip_flex/ext_ - Knee_abd/add_ |  |  | | 4.5 | | | 12.5 | | | |  | | | 1.6 | | 4.4 | | |  |  |
|  |  |  | |  | | |  | | | |  | | |  | |  | | |  |  |
| Hip_abd/add_ - Knee_flex/ext_ |  |  | | 7.4 | | | 20.5 | | | |  | | | 0.7 | | 1.9 | | |  |  |
|  |  |  | |  | | |  | | | |  | | |  | |  | | |  |  |
| Hip_abd/add_ - Knee_abd/add_ |  |  | | 3.7 | | | 10.3 | | | |  | | | 1.2 | | 3.3 | | |  |  |
|  |  |  |  | | |  | | |  |  | | |  | | | |  |  |  |  |
