# Supplementary material for: Gait and Neuromuscular Changes Are Evident in Some Masters Club Level Runners 24-h After Interval Training Run
Source: Front Sports Act Living. 2022 Jun 2;4:830278. doi: 10.3389/fspor.2022.830278 (PMC9201250; doi:10.3389/fspor.2022.830278)
Supplement: Supplementary file 4 [file Table_4.DOCX]

| Supplementary Digital Content 4. Standard error of measurement (SEM) and minimum detectable change (MDC) values maximum voluntary contraction (MVC, Newtons) of the knee extensors, voluntary activation percentage (VA%), and for quadriceps resting twitch potential (Q_tw_,_pot,_, Newtons). | | | | |  |
| --- | --- | --- | --- | --- | --- |
|  |  |  | |  | |
|  | SEM | | MDC | |  |
|  |  | |  | |  |
| MVC | 22.5 | | 62.4 | |  |
| Q_tw_,_pot_ (N) | 13.2 | | 24.3 | |  |
| VA% | 1.8 | | 5.0 | |  |
